# Oxysterols as lipid mediators: Their biosynthetic genes, enzymes and metabolites

**DOI:** 10.1016/j.prostaglandins.2019.106381

**Published:** 2020-04

**Authors:** William J. Griffiths, Yuqin Wang

**Affiliations:** Swansea University Medical School, ILS1 Building, Singleton Park, Swansea, SA2 8PP Wales, UK

**Keywords:** Hydroxycholesterol, Dihydroxycholesterol, Epoxycholesterol, G protein-coupled receptor, Epstein Barr virus induced gene 2, Smoothened, Hedgehog signaling, Nuclear receptor, Liver X receptor

## Abstract

There is growing evidence that oxysterols are more than simple metabolites in the pathway from cholesterol to bile acids. Recent data has shown oxysterols to be ligands to nuclear receptors and to G protein-coupled receptors, modulators of *N*-methyl-d-aspartate receptors and regulators of cholesterol biosynthesis. In this mini-review we will discuss the biosynthetic mechanisms for the formation of different oxysterols and the implication of disruption of these mechanisms in health and disease.

## Highlights

• Pathways of oxysterol biosynthesis.
• Pathways of oxysterol metabolism.
• Oxysterols as bioactive molecules.
• Disorders of oxysterol metabolism.

## 1 Introduction

Oxysterols are oxidised forms of cholesterol or of its precursors [1,2]. They are early intermediates in the metabolism of cholesterol to bile acids. Oxysterols possess a wide range of biological properties acting as ligands towards nuclear receptors [[3], [4], [5], [6]] and to G protein-coupled receptors (GPCRs) [[7], [8], [9], [10], [11], [12]] and modulators of *N*-methyl-D-aspartate receptors (NMDARs) [13]. Oxysterols also bind to INSIG (insulin induced gene) tethering SCAP (SREBP cleavage-activating protein) and SREBP-2 (sterol regulatory-element binding protein-2) in the endoplasmic reticulum (ER), preventing transport of SREBP-2 to the Golgi for processing to its active form as a transcription factor for the genes of the cholesterol biosynthesis pathway [14].

In vertebrates, oxysterols are mostly synthesised in enzyme catalysed reaction [15] but can also be formed via non-enzymatic routes [[16], [17], [18]]. The main enzymes responsible for introducing hydroxy groups to generate oxysterols are members of the cytochrome P450 (CYP) superfamily, while further oxidation may proceed by additional action of CYPs or hydroxysteroid dehydrogenase (HSD) enzymes. Cholesterol 25-hydroxylase (CH25 H) is an exception in that it is a not a CYP but member of a family of enzymes that use diiron co-factors to catalyse hydroxylation [19].

## 2 Main pathways of oxysterol biosynthesis

Oxysterol biosynthesis can be divided into a number of different pathways depending on the site of the initial oxidation, these pathways may overlap and lead ultimately to bile acid formation.

### 2.1 7α-Hydroxylase pathway

This embraces the “neutral” or “classical” pathway of bile acid biosynthesis and intermediates include 7α,(25R)26-dihydroxycholesterol (7α,26-diHC, unless specifically stated stereochemistry is assumed to be 25R, also known as 7α,27-dihydroxycholesterol) a ligand to the GPCR183 (Epstein-Barr virus induced gene 2, EBI2) [7,8].

**Fig. 1:**
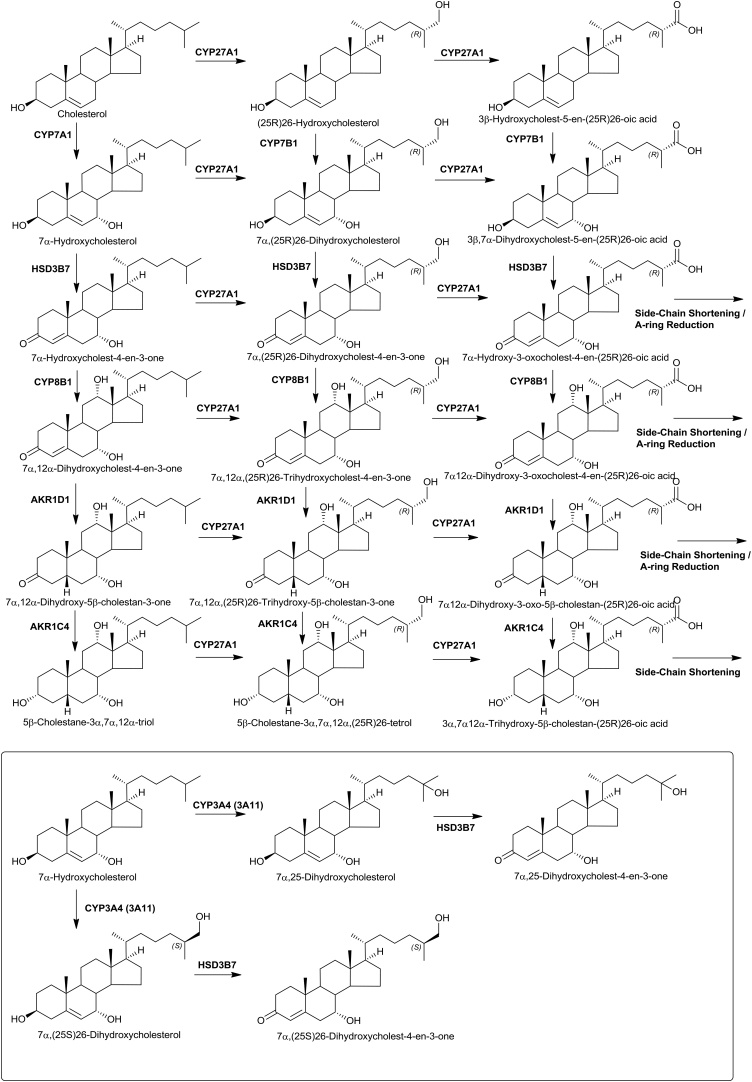
7α-Hydroxylase and (25R)26-hydroxylase pathways of oxysterol biosynthesis. The 7α-hydroxylase pathway starts with 7α-hydroxylation of cholesterol and the (25R)26-hydroxylase pathway begins with (25R)26-hydroxylation of cholesterol.

The first step of the pathway is 7α-hydroxylation of cholesterol by CYP7A1, the transcript of which is almost exclusively expressed in liver (Supplemental Table S1) [[20], [21], [22]], to give 7α-hydroxycholesterol (7α−HC). This is the rate determining step in the “neutral” pathway of bile acid biosynthesis. 7α−HC may be (25R)26-hydroxylated then (25R)26-carboxylated by CYP27A1, expressed in multiple organs [[21], [22], [23]], to 7α,26-diHC and 3β,7α-dihydroxycholest-5-en-(25R)26-oic acid (3β,7α-diHCA), respectively (Fig. 1). Alternatively, 7α−HC may be oxidised by ubiquitously expressed HSD3B7 [21,22,24] to 7α-hydroxycholest-4-en-3-one (7α−HCO). 7α−HCO is a ligand to the pregnane X receptor (PXR), a member of the nuclear receptor superfamily [25]. Another route for 7α−HC metabolism is by CYP3A4 in human, and CYP3A11 in mouse, to the most potent EBI2 agonist 7α,25-dihydroxycholesterol (7α,25-diHC) and also to a lesser extent 7α,(25S)26-dihydroxycholesterol (7α(25S)26-diHC) [26]. PXR is activated by 7α−HCO [25] and regulates the expression of CYP3A4 in human, 3A11 in mouse [27]. HSD3B7 requires a 7α-hydroxy group in its substrates and can oxidise 7α,26-diHC, 7α,25-diHC and 3β,7α-diHC to their respective 3-oxo-4-ene equivalents 7α,(25R)26-dihydroxycholest-4-en-3-one (7α,26-diHCO), 7α,25-dihydroxycholest-4-en-3-one (7α,25-diHCO) and 7α-hydroxy-3-oxocholest-4-en-(25R)26-oic acid (7αH,3O-CA). The ultimate metabolite of the “neutral” pathway is predominantly cholic and to a lesser extent chenodeoxycholic acid. In cholic acid biosynthesis CYP8B1 introduces a 12α-hydroxy group into 7α−HC and 7α−HCO to give 7α,12α-dihydroxycholesterol (7α,12α-diHC) and mostly 7α,12α-dihydroxycholest-4-en-3-one (7α,12α-diHCO) [28,29].

In the “classical” bile acid biosynthesis pathway 7α,12α-diHCO becomes reduced by aldoketo reductase 1D1 (AKR1D1) then AKR1C4 to 5β-cholestane-3α,7α,12α-triol which is a substrate for CYP27A1 oxidation first to the 3α,7α,12α,(25R)26-tetrol and then the 3α,7α,12α-trihydroy-5β-cholestan-(25R)26-oic acid which is then side-chain shortened to cholic acid in the peroxisome [[30], [31], [32]]. Alternatively, 7α,12α-diHC, 7α,12α-diHCO, 7α,26-diHC, 7α,26-diHCO can cross into the (25R)26-hydroxylase pathway and 7α,25-diHC and 7α,25-diHCO fall into the “25-hydroxylase” pathways as described below (Fig. 1).

The expression of *CYP7A1*, the gene encoding the rate-limiting step of the “neutral” pathway of bile acid biosynthesis, and of *CYP8B1*, is regulated by the farnesoid X receptor (FXR), activated by cholic and chenodeoxycholic acids [33,34]. FXR activates another nuclear receptor, short hetrodimeric partner (SHP), which binds and inhibits a third nuclear receptor, liver receptor homologue 1 (LRH-1), which activates the expression of *CYP7A1* and *CYP8B1* [15]. The net result of activation of FXR by bile acids is inhibition of *CYP7A1* expression and their own biosynthesis. In mouse *CYP7A1* expression is also regulated by the liver X receptor (LXR) [35].

### 2.2 25-Hydroxylase pathway

**Fig. 2:**
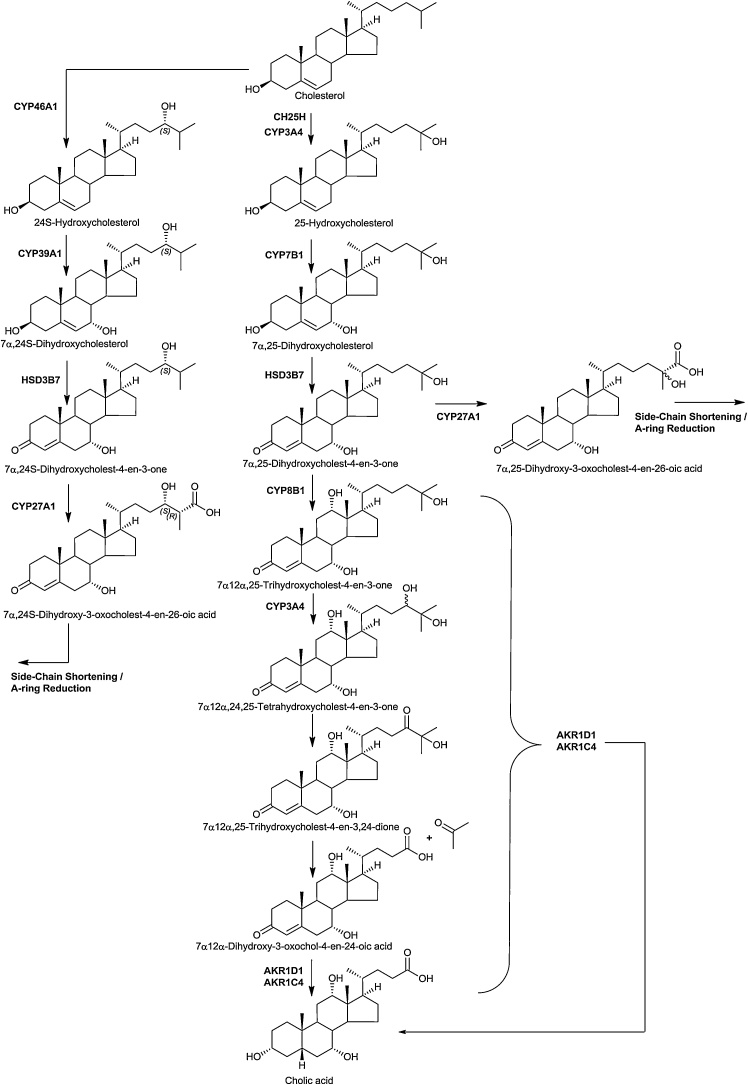
24S-Hydroxylase and 25-hydroxylase pathways of oxysterol biosynthesis.

The “25-hydroxylase” pathway is considered to start with 25-hydroxylation of cholesterol by CH25H (Fig. 2) [19]. *CH25H* is located on chromosome 10 in man, while *Ch25h* is located on chromosome 19 in mouse. It is an interferon-stimulated gene expressed in activated immune cells [[36], [37], [38]]. The enzyme product 25-hydroxycholesterol (25−HC) supresses cholesterol synthesis and is a ligand to the LXRs and to INSIG [3,14], suppresses interlukin-1 driven inflammation [39,40] and inhibits viral infection in a paracrine manner [38,41]. There is only one report of CH25H deficiency in human, but the disorder presented with combined deficiency of the adjacent gene lysosomal acid lipase (*LIPA*), and the first patient was initially diagnosed with Wolman disease (infantile onset lysosomal acid lipase deficiency) [42]. Patients presented with susceptibility to abyss in response to Bacillus Calmette–Guérin (BCG) vaccination [42]. CYP3A4 can also act as an alternative cholesterol 25-hydroxylase, accounting for production of 25−HC in the absence of CH25H [43].

25−HC is metabolised by CYP7B1 to the EBI2 ligand 7α,25-diHC [7,44]. Deficiency in CYP7B1 leads to oxysterol 7α-hydroxylase deficiency in infants [45] and hereditary spastic paraplegia type 5 (SPG5) in adults [46], although the SPG5 phenotype is believed to be a consequence of disruption of the (25R)26-hydroxylase pathway rather than the 25-hydroxylase pathway. *CYP7B1* is expressed in many tissues [21]. By virtue of the presence of a 7α-hydroxy group, 7α,25-diHC is a substrate for HSD3B7 and can be converted to 7α,25-diHCO. The exact routes for metabolism of 7α,25-diHCO are still to be fully established. One pathway is further oxidation, initially by CYP8B1 at C-12, then at C-24 first to an alcohol, by CYP3A4 in man and CYP3A11 in mouse [27], then to a carbonyl followed by elimination of acetone with subsequent bile acid formation as suggested by Duane et al [47]. Reduction and oxidation, presumably by AKR1D1 and AKR1C4 may precede before [32] or after 24-hydroxylation [48]. We have recently uncovered an unexpected 25-hydroxylated acid, 7α,25-dihydroxy-3-oxocholest-4-en-26-oic acid (7α,25-diH,3O-CA) in human plasma and cerebrospinal fluid (CSF) [49]. Interestingly, its level is reduced in CSF from patients suffering from Alzheimer’s disease [48]. Correlation analysis of cholesterol metabolites found in CSF suggests that 7α,25-diHCO, 7α,25-diH,3O-CA and 7α-hydroxy-3-oxochol-4-en-24-oic acid (7αH,3O-Δ^5^-BA) constitute a pathway towards chenodeoxycholic acid [48].

### 2.3 24S-Hydroxylase pathway

Cholesterol is metabolised to 24S-hydroxycholesterol (24S−HC) by CYP46A1 (Fig. 2). 24S−HC is a ligand to both LXRs and INSIG [3,14]. Unlike cholesterol, 24S−HC can cross the blood brain barrier (BBB) and represents a route to removal of cholesterol from brain [50], besides regulating cholesterol synthesis through INSIG and the SREBP-2 pathway. In human and mouse CYP46A1 is expressed in brain [51]. There is some minor expression of the gene in other tissues including testis and ovary [21,22], however, at least in mouse, 24S−HC found in the circulation is derived predominantly from brain [52]. 24S−HC is 7α-hydroxylated to 7α,24S-diHC by CYP39A1 [53]. The gene is expressed mostly in liver but also in brain [21,22]. 7α,24S-diHC can be oxidised by HSD3B7 to 7α,24S-dihydroxycholest-4-en-3-one (7α,24S-diHCO) which is found in mouse brain and plasma [26,54]. 7α,24S-diHC has been found in human plasma [55]. 7α,24S-diHC and 7α,24S-diHCO may provide substrates for CYP27A1 and crossover to the (25R)26-hydroxylase pathway (see below). 7α,24-Dihydroxy-3-oxocholest-4-en-26-oic acid (7α,24-diH,3O-CA) has been identified in human plasma and CSF [48,49]. As discussed below 7α,24S-diH,3O-CA undergoes side-chain shortening in the peroxisome to 7αH,3O-Δ^5^-BA which can then be reduced by AKR1D1 and AKR1C4 to chenodeoxycholic acid.

### 2.4 (25R)26-hydroxylase pathway

The (25R)26-hydroxylase pathway begins with (25R)26-hydroxylation of cholesterol by CYP27A1 to give (25R)26-hydroxycholesterol (26−HC, 25R-stereochemistry is assumed unless indicated otherwise, also called 27-hydroxycholesterol) which can be further oxidised to 3β-hydroxycholest-5-en-(25R)26-oic acid (3β−HCA) by the same enzyme (Fig. 1). A defect in the enzyme CYP27A1 leads to the disorder cerebrotendinous xanthomatosis (CTX) [56], where this pathway is inactive. In contrast to human, deficiency in CYP27A1 enzyme activity in mouse, as revealed by the *Cyp27a1* knock-out (*Cyp27a1-/-*) animal, leads to only a mild phenotype [57]. There are two branches of the (25R)26-hydroxylase pathway, the first starts from 26−HC with 7α-hydroxylation give 7α,26-diHC and the second from 3β−HCA with 7α-hydroxylation to give 3β,7α-diHCA, both reactions being catalysed by CYP7B1. The pathway from 3β−HCA is the “acidic” or “alternative” pathway of bile acid biosynthesis [15]. 26−HC, 3β−HCA and 3β,7α-diHCA can act as LXR ligands [[58], [59], [60], [61]], while 26−HC will also repress cholesterol synthesis by binding to INSIG [14]. 26−HC has been shown to be a selective estrogen receptor modulator (SERM) and has been implicated with breast cancer [62,63]. The EBI2 ligand 7α,26-diHC can be deactivated by HSD3B7 to 7α,26-diHCO, or by CYP27A1 to 3β,7α-diHCA. 7α,26-diHCO can be oxidised further by CYP27A1 to 7αH,3O-CA. The two branches of the 26-hydroxylase pathway converge at 7αH,3O-CA with HSD3B7 oxidation of 3β,7α-diHCA. Interestingly, 3β−HCA and 3β,7α-diHCA are both ligands to LXR but have opposite effects on oculomotor neuron survival during development [59]. Reduction in the A-ring of 7αH,3O-CA by AKR1D1 and AKR1C4 can proceed before or after side-chain shortening in the peroxisome to ultimately lead to chenodeoxycholic acid [32].

## 3 Peroxisomal side-chain shortening

Peroxisomes are organelles important for the β-oxidation and side-chain shortening of bile acids. They also serve to conjugate newly synthesised bile acids with glycine or taurine. Patients suffering from Zellweger syndrome, which results from an absence of functional peroxisomes, show an accumulation of C_27_ bile acids [64]. Zellweger syndrome is an autosomal recessive disorder and presents with impaired brain development, degeneration of central nervous system myelin and enlarged liver. Children with this disorder usually die within the first year reflecting the essential functions of the peroxisome [65].

Cholestenoic and cholestanoic acids can be included in the oxysterol family hence their side-chain shortening reactions are discussed here. In the following section we will describe side-chain shortening for 7αH,3O-CA but the same reaction sequence is applicable to A-ring reduced and also to 12α-hydroxy metabolites.

**Fig. 3:**
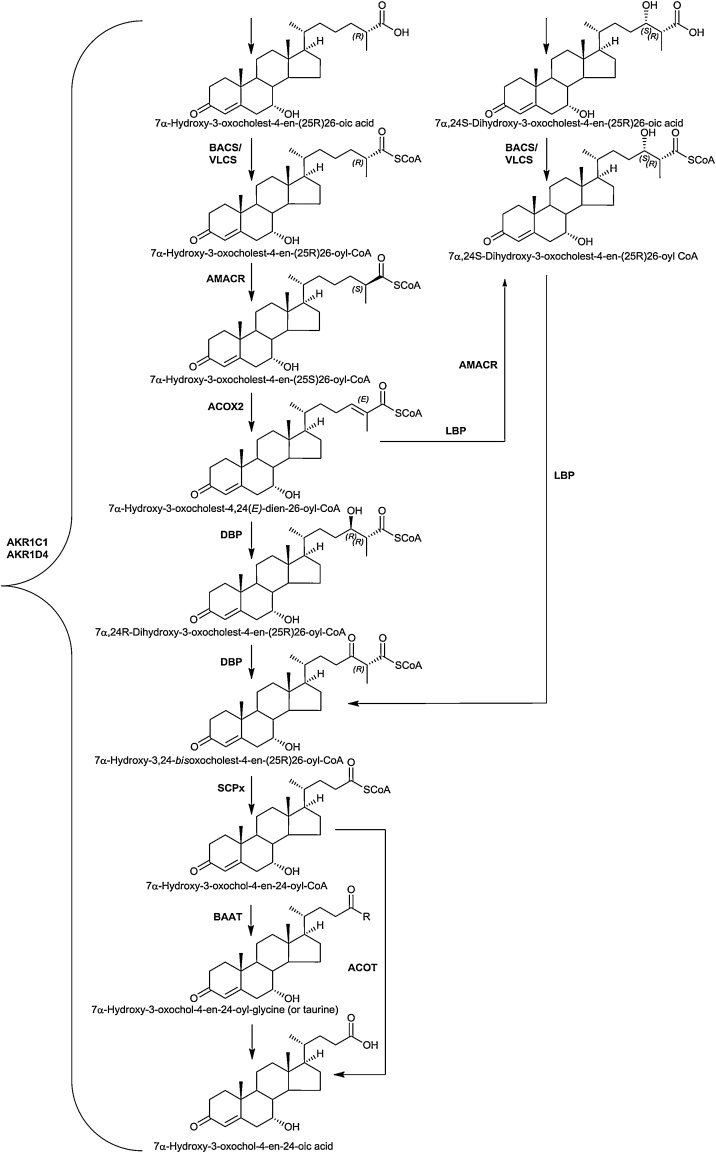
Peroxisomal side-chain shortening.

The first step is to form a CoA-thioester, this is achieved by bile acid CoA ligase (or synthetase, BACS, SLC27A5) or by very-long chain acyl-CoA synthetase (VLCS, SLC27A2, Fig. 3). BACS is a microsomal protein mostly expressed in liver, while VLCS is expressed mostly in liver and kidney and is present in the ER and peroxisome [21,22]. The ABC transporter protein ABCD3 is required to transport C_27_-bile acyl-CoAs into the peroxisome. When the acyl-CoA is derived from the “acidic” pathway it will have 25R stereochemistry. However, for peroxisomal side-chain shortening it is first necessary to convert this to the 25S-epimer. This is achieved by the broadly expressed α-methylacyl-CoA racemase (AMACR) [21,22], mutations in which can lead to AMACR deficiency [66,67]. Next, a double bond with *E* stereochemistry is introduced between C-24 and C-25 by acyl-CoA oxidase 2 (ACOX2). Patients with ACOX2 deficiency were discovered in 2016 by Vilarinho et al showing liver fibrosis, ataxia and cognitive impairment [68]. The *E* double bond is next hydrolysed by the hydratase activity of D-bifunctional protein (DBP, MFE2, HSD17B4) to give the 24R-hydroxy-(25R)26-acyl-CoA, which is then oxidised to the 24-oxo-(25R)26-acyl-CoA by the HSD activity of DBP. Sterol carrier protein x (SCPx, SCP2) catalyses the last step in β-oxidation to give the C_24_-acyl-CoA which can be conjugated with glycine or taurine by bile acyl-CoA : amino acid N-acyl transferase (BAAT) or hydrolysed to the C_24_ carboxylic acid by peroxisomal acyl-CoA thioesterase (ACOT) [69,70]. Patients with deficiencies in DBP [71], SCPx [72] and BAAT [73] have been discovered.

When initial hydroxylation of cholesterol is by CYP46A1 to give 24S−HC, the down-stream C_27_ acid prior to peroxisomal processing has 24S-hydroxy-(25R)26-carboxylate stereochemistry. This stereochemistry in the subsequent acyl-CoA thioester provides a substrate for HSD activity of L-bifunctional protein (LBP, MFP1, enoyl-CoA hydratase and 3-hydroxyacyl CoA dehydrogenase, EHHADH) to generate 24-oxo-(25R)26-acyl-CoA for further processing by SCPx. In mouse 24R−HC is present in plasma [52], metabolism to the down-stream 24R-hydroxy-(25R)26-acyl-CoA will provide a substrate for the HSD activity of DBP to generate the 24-oxo-(25R)26-acyl-CoA which can then undergo side-chain shortening.

Bile acids can be formed in the absence of CYP27A1. This is evident in patients with CTX and in the *Cyp27a1-/-* mouse [26,56]. In the *Cyp27a1-/-* mouse there is some sterol (25S)26-hydroxylase activity. This has been suggested to be via CYP11A1 in mouse and CYP3A4 in man [26,27]. We have suggested a pathway to C_27_-(25S)26-acyl-CoA’s which are substrates for ACOX2 [26].

## 4 Other pathways of oxysterol biosynthesis and metabolism

### 4.1 7-Oxocholesterol and 7β-hydroxycholesterol pathways

#### 4.1.1 Smith-Lemli-Opitz syndrome

**Fig. 4:**
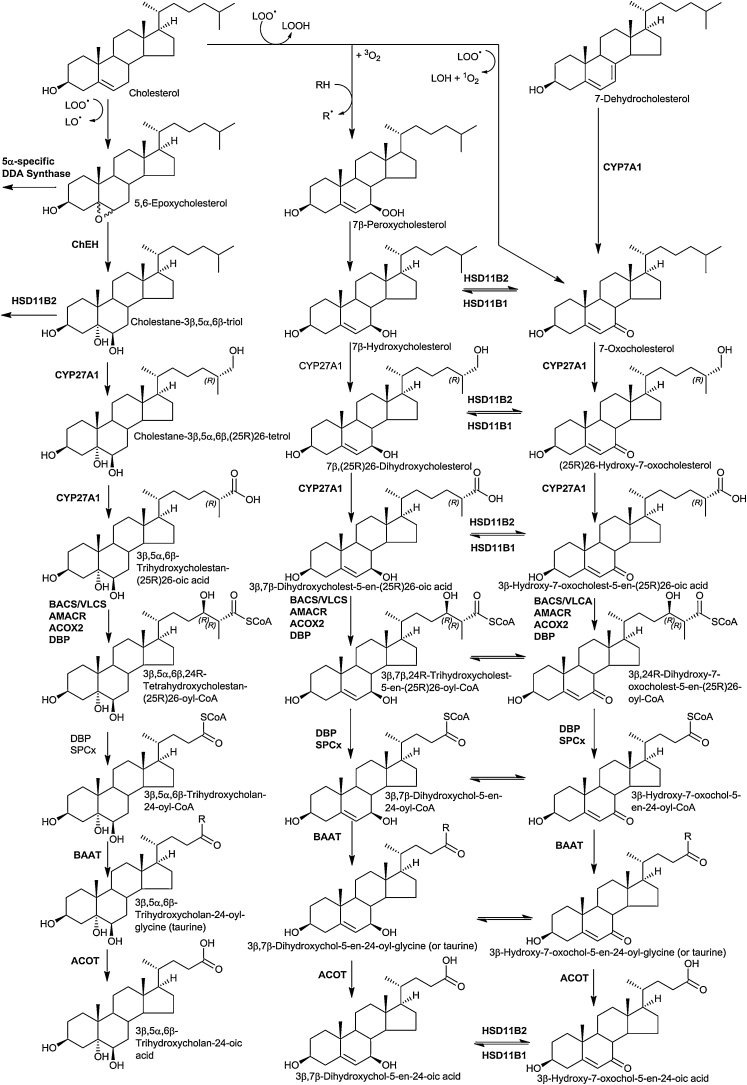
Cholesterol epoxide hydrolase, 7β-hydroxy and 7-oxo pathways of oxysterol biosynthesis.

7-Oxocholesterol (7−OC) can be formed from 7-dehydrocholesterol (7-DHC) by CYP7A1 [74] (Fig. 4). It can then be reduced to 7β-hydroxycholesterol (7β−HC) by HSD11B1, the same enzyme that reduces cortisone to cortisol [[75], [76], [77]]. HSD11B2 catalyses the reverse reaction [12]. We have shown that in the disorder, Smith-Lemli-Opitz syndrome (SLOS) where 7-DHC is abundant, on account of a defect in 7-dehdrocholesterol reductase (DHCR7), 26-hydroxy-7-oxocholesterol (26H,7O-C) and 25-hydroxy-7-oxocholesterol (25H,7O-C) are evident in plasma, as are their 7β-reduced forms, presumably formed by HSD11B1 from the 7-oxo precursors [[78], [79], [80], [81]]. 26H,7O-C, 25H,7O-C and 7β,26-dihydroxycholesterol (7β,26-diHC, also called 7β,27-dihydroxycholesterol) will bind to the extracellular cysteine rich domain (CRD) of Smoothened (SMO), the GPCR involved in the Hedgehog (Hh) signalling pathway and activate the pathway [10,12]. Disturbed Hh signalling is implicated in the SLOS phenotype, which is a disorder presenting with dysmorphology [82]. Data from analysis of plasma from people with SLOS and from amniotic fluid of affected pregnancies shows that 26H,7O-C and 7β,26-diHC both cross into the “acidic” pathway of bile acid biosynthesis with formation of 3β-hydroxy-7-oxocholest-5-en-26-oic (3βH,7O-CA) and 3β,7β-diHCA (Fig. 4). 3βH,7O-CA can also modulate Hh signalling by binding to the CRD of SMO [78,79], while 3β,7β-diHCA is a ligand to the nuclear receptor RAR-related orphan receptor gamma t (RORγt) [5]. In the absence of a 7α-hydroxy group, 7β-hydroxy and 7-oxosterols cannot undergo transformation of the 3β-hydroxy-5-ene structure to the 3-oxo-4-ene by HSD3B7, hence the 3β-hydroxy-7-oxo-5-ene and 3β,7β-dihydroxy-5-ene structures are maintained through bile acid biosynthesis to the 3β-hydroxy-7-oxochol-5-en-24-oic (3βH,7O-Δ^5^-BA) and 3β,7β-dihydroxychol-5-en-24-oic (3β,7β-diH-Δ^5^-BA) acids and their glycine and taurine conjugates [55,78,79]. The 7β-hydroxy group can be conjugated with *N*-acetylglucosamine (GlcNAc) in a reaction catalysed by UGT3A1 [83] and bile acids conjugated with GlcNAc and glycine or taurine and sulphuric acid have been identified in SLOS patients [79].

#### 4.1.2 Lysosomal storage disorders; Niemann pick disease types B and C and Wolman’s disease

**Fig. 5:**
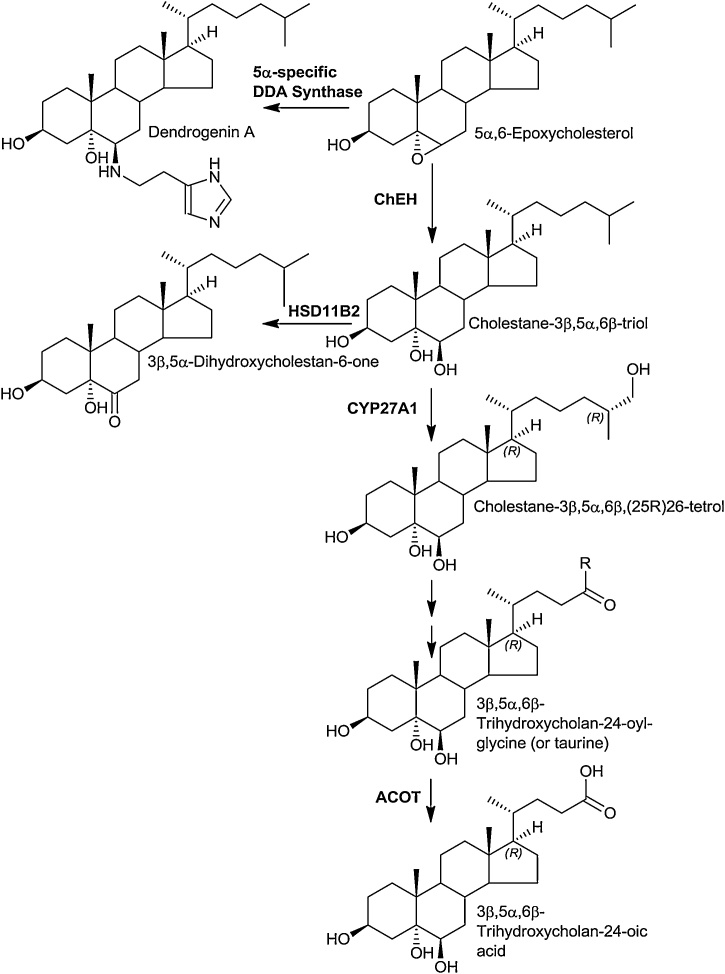
Metabolism of 5α,6-epoxycholesterol.

The lysosomal storage disorder Niemann Pick (NP) disease type B results from mutations in the sphingomyelin phosphodiesterase 1 (*SMPD1*) gene, while NP disease types C1 and C2 result from mutations in the *NPC1* and *NPC2* genes, respectively. Mutations in all three proteins lead to a similar clinical phenotype with accumulation of non-esterified cholesterol in late endosomes/lysosomes [84]. The NPC1 and NPC2 proteins are involved in export of non-esterified cholesterol from the lumen of late endosomes/lysosomes. Within late endosomes/lysosomes the soluble NPC2 protein shuttles cholesterol to the membrane bound NPC1 protein, avoiding contact of the sterol with the aqueous environment. The mechanism by which NPC1 transports cholesterol from the late endosomes/lysosomes to the ER and plasma membrane has yet to be established [84]. A further protein in lysosomes is lysosomal acid lipase (LAL), coded by the *LIPA* gene on chromosome 10 in human, which hydrolyses cholesterol esters taken up in LDL by receptor mediated endocytosis into endosomes, making non-esterified cholesterol available for NPC transport [85]. In infants LAL deficiency presents as Wolman’s disease, primarily characterised by accumulation of cholesterol esters and triglycerides in liver spleen and lymph nodes [85]. Diagnostic features of both NPB and NPC are elevated plasma levels of 7−OC and cholestane-3β,5α,6β-triol (3β,5α,6β-triol) [86]. 3β,5α,6β-Triol is generated by hydrolysis of 5,6-epoxycholesterol (5,6-EC) by the enzyme cholesterol epoxide hydrolase (ChEH) [87]. ChEH is heterodimer of two other enzymes, 3β-hydroxysterol-Δ^8^-Δ^7^-isomerase (D8D7I, EBP, emopamil-binding protein) and DHCR7. An enzyme converting cholesterol to 5,6-EC has yet to be established and it is likely that it is formed in lysosomal storage disorders by non-enzymatic oxidation in late endosomes/lysosomes (Fig. 4). An alternative metabolic route for metabolism of 5α,6-EC, but not 5β,6-EC, is conjugation with histamine to give dendrogenine A (DDA), in a reaction catalysed by DDA synthase (Fig. 5). DDA shows tumour suppressor properties [88,89]. Interestingly, DDA is a partial agonist of LXR triggering autolysosome formation [88].

There appear to be two routes for metabolism of 3β,5α,6β-triol, one route is metabolism to 3β,5α,6β-trihydroxycholan-24-oic acid (3β,5α,6β-triHBA) and its conjugates [55,83,90,91], the other, oxidation by HSD11B2 to the oncometabolite 3β,5α-dihydroxycholestan-6-one (3β,5α-diHC-6O, 6-oxo-cholestan-3β,5α-diol, OCDO, Fig. 5) [6]. 3β,5α-diHC-6O stimulates cell growth by binding to the glucocorticoid receptor [6]. By analysis of plasma from patients with NPB, NPC and Wolman’s disease, *LIPA* deficiency presenting in infants, where 3β,5α,6β-triol is abundant, a metabolic pathway where 3β,5α,6β-triol is oxidised by CYP27A1 and proceeds along the same route as the “acidic” pathway has been established [55].

As in SLOS, elevated plasma levels of 7−OC and 7β−HC are found patients with NPB, NPC and Wolman’s disease and become metabolised in pathways identical to those described for SLOS [55,91]. 7-DHC is not elevated in these lysosomal storage disorders and it is highly likely that 7−OC and 7β−HC are derived via non-enzymatic oxidation of cholesterol [55]. It is of interest, that as far back as 2001 Alvelius et al described unusual 7-oxo-bile acids in a patient with NPC [92], while as early as 1994 Natowski and Evans described abnormal bile acids in urine from SLOS patients [93]. On account of its formation through non-enzymatic oxidation the metabolism of 7−OC has been of interest for decades [94,95].

### 4.2 24S,25-Epoxycholesterol synthesis and metabolism

**Fig. 6:**
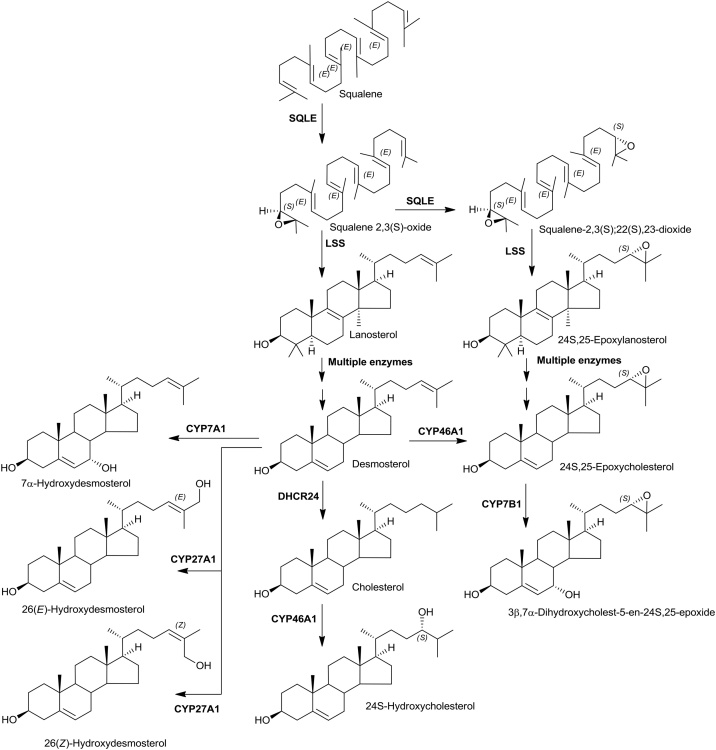
Biosynthesis of 24S,25-epoxycholesterol and of other oxysterols from desmosterol.

24S,25-Expoxycholesterol (24S,25-EC) can be formed via a shunt of the mevalonate pathway, using the same enzymes as in cholesterol biosynthesis with the exception of 24-dehydrocholesterol reductase (DHCR24), which is not utilised in the shunt pathway (Fig. 6) [96,97]. In the shunt pathway, squalene epoxidase (SQLE) introduces a second epoxy group into squalene to give squalene-2,3(S);22(S),23-dioxide, rather than the normal squalene-2,3(*S*)-oxide. Squalene-2,3(S);22(S),23-dioxide is then cyclised by lanosterol synthase (LSS) and metabolised in parallel to lanosterol in the Block pathway to give 24S,25-EC. An alternative route to biosynthesis of 24S,25-EC is by epoxidation of the C24 - C25 double bond in desmosterol by CYP46A1 [98]. 24S,24-EC has been found to be elevated in brain and plasma of mice over expressing the human CYP46A1 enzyme [99]. This is compatible with formation of 24S,24-EC from desmosterol via CYP46A1 catalysis in brain, but could also be explained by an increased passage of metabolites through the mevalonate pathway, and its shunt, as a consequence of enhanced cholesterol removal by CYP46A1 metabolism and a reduction in negative-feedback of the pathway by cholesterol [100].

24S,25-EC is underreported in oxysterol analysis because of difficulties in its detection by mass spectrometry. It is a bioactive molecule, acting as a ligand to the LXRs [3], to INSIG [14] and has recently been shown to bind to SMO and activate the Hh pathway [12,101]. Theofilopoulos et al have shown that 24S,25-EC promotes midbrain motor neuron neurogenesis through activation of the LXR receptors [99,102]. The likely metabolic route of 24S,25-EC is 7α-hydroxylation to 3β,7α-dihydroxycholest-5-en-24S,25-epoxide by CYP7B1 [54,103].

### 4.3 22R-Hydroxylase pathway

**Fig. 7:**
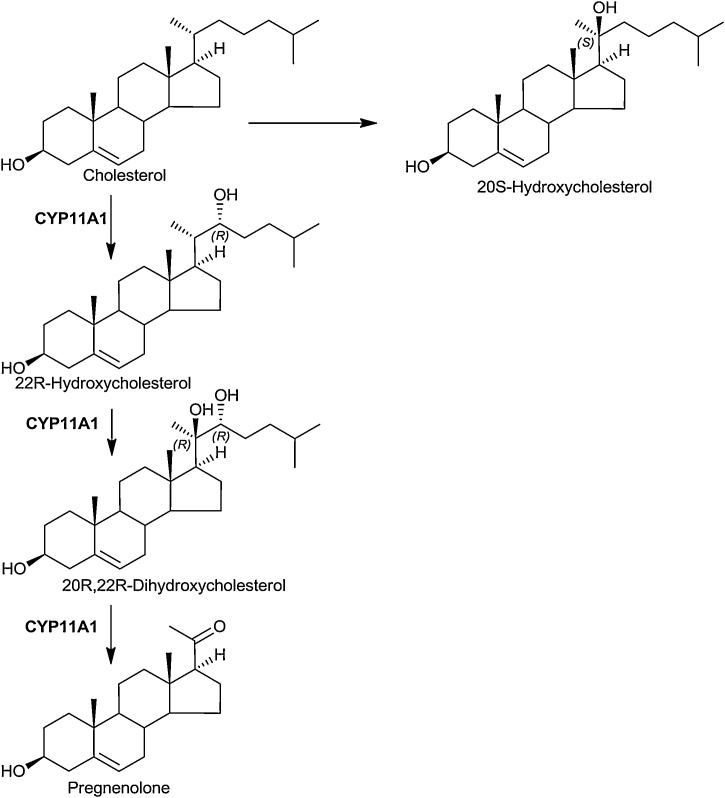
22R-Hydroxylase pathway.

The first step of steroid hormone biosynthesis is the 22R-hydroxylation of cholesterol by CYP11A1 (P450scc) to give 22R-hydroxycholesterol (22R−HC). This is followed by a second hydroxylation by the same enzyme to give 20R,22R-dihydroxycholesterol (20R,22R-diHC) which undergoes side-chain cleavage to pregnenolone catalysed by the same enzyme (Fig. 7). Both 22R−HC and 20R,22R-diHC are found in mouse plasma [26]. Recent unpublished data from our laboratory shows that 20R,22R-diHC is particularly abundant in plasma from human mothers’ umbilical cord blood. This is perhaps not surprising as CYP11A1 is highly expressed in placenta [21].

### 4.4 20S-Hydroxycholesterol pathway

20S-Hydroxycholesterol (20S−HC) is the most potent oxysterol agonist towards the Hh signalling pathway. It binds to the CRD of SMO [9]. 20S−HC is an elusive oxysterol with few definitive identifications [104]. However, Lin et al identified 20S−HC in rat brain and in human placenta [105]. We have similarly found 20S−HC to be in mouse brain and also human placenta [106]. It is not clear which enzyme generates 20S−HC, the KEGG pathway https://www.genome.jp/kegg-bin/show_pathway?map00140+C05501 implicates CYP11A1 but the literature does not appear to be in accord with this.

### 4.5 Oxysterols derived from cholesterol precursors

As discussed for 24S,25-EC above, oxysterols can be generated from cholesterol precursors as well as cholesterol itself.

#### 4.5.1 Desmosterol

Björkhem and colleagues have created a mouse model of the human disorder desmosterolosis, where DHCR24 is dysfunctional [107,108]. Surprisingly, the mice had only a mild phenotype [107]. Analysis of plasma from the *Dhcr24-/-* mouse revealed the presence of 26-hydroxydesmosterol (26-HD, also called 27-hydroxydesmosterol) but not 7α-hydroxydesmosterol (7α-HD), although desmosterol is a substrate for both CYP27A1 and CYP7A1 (Fig. 6) [108]. More recent studies have identified both the (*Z*) and (*E*) isomers of 26-HD in mouse brain [54] and 7α-HD in the circulation of the *Cyp27a1-/-* mouse where CYP7A1 is up-regulated [26]. While desmosterol acts as an LXR ligand [108,109], neither of the 26-HD isomers are active, providing a route to deactivation of desmosterol [110].

#### 4.5.2 7-DHC and 8-DHC

**Fig. 8:**
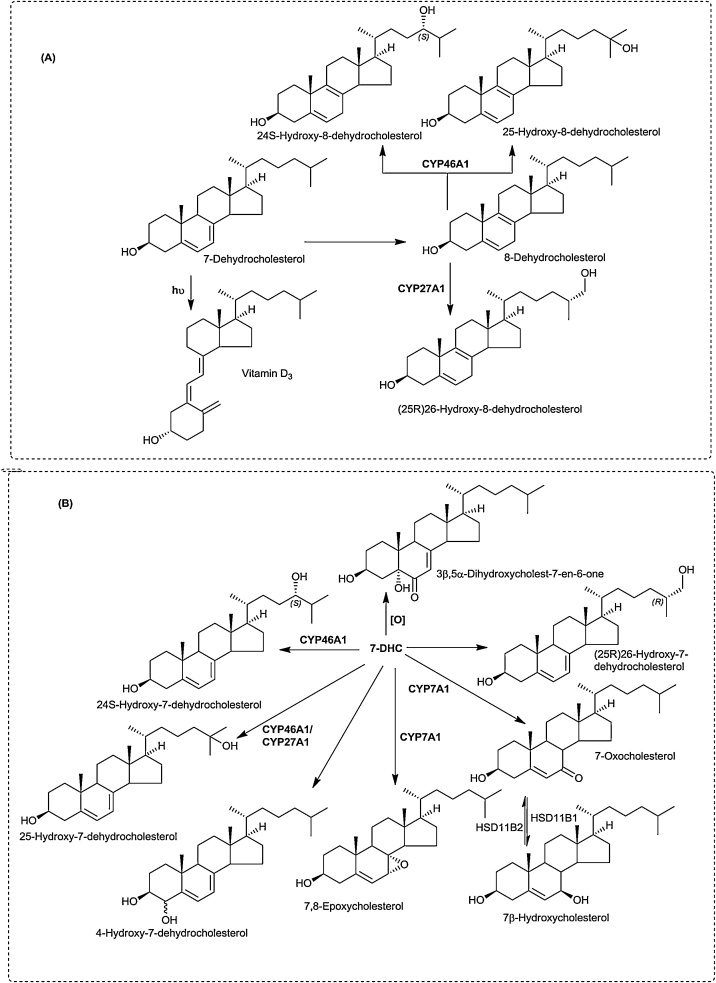
Oxysterols derived from 7-DHC.

Besides the pathway discussed above where CYP7A1 converts 7-DHC to 7−OC, there are other pathways from 7-DHC, both enzymatic, and non-enzymatic, to oxysterols (Fig. 8) [74,98,111]. 7-DHC is elevated in the disorder SLOS where DHCR7 is deficient. 7-DHC will isomerise to 8-DHC and metabolites of both these sterols are evident in plasma from SLOS subjects. In recent studies we have identified enzymatically derived 7,8-epoxycholesterol (7,8-EC, 3β-hydroxycholest-5-en-7,8-epoxide), 24- or 25-hydroxy-8-dehydrocholesterol (24H-8-DHC or 25H-8-DHC) and 26-hydroxy-8-dehydrocholesterol (26H-8-DHC) in plasma from SLOS patients (Fig. 8) [112,113]. Others have identified 26-hydroxy-7-dehydrocholesterol (26H-7-DHC), 26H-8-DHC and 4α- and 4β-hydroxy-7-dehydrocholesterol (4αH-7-DHC, 4βH-7-DHC) in SLOS plasma [114,115]. Both 26H-7-DHC and 25-hydroxy-7-dehydrocholesterol (25H-7-DHC) were found to be partial activators of LXRs [114,115].

7-DHC is susceptible to free radical oxidation reactions which may proceed *in vivo* and *ex vivo* [111,116]. A prominent 7-DHC metabolite formed in fibroblasts from SLOS patients is 3β,5α-dihydroxycholest-7-en-6-one. This oxysterol is present in plasma from SLOS patients [113]. It blocks Hh signalling by binding to SMO at a binding site distinct from other oxysterols [11].

## 5 Discussion

Oxysterols are formed through a myriad of enzyme catalysed and non-enzymatic reactions. They have a very wide range of properties including acting as ligands to nuclear receptors including the LXRs [3,[58], [59], [60], [61],^88^], PXR [25], RORγt [5], the estrogen receptors [4] and the glucocorticoid receptor [6]. They bind to INSIG and inhibit the processing of SREBP-2 to its active form as a transcription factor [14] and they also bind to and modulate the NMDARs [13]. More recently, oxysterols have been shown to be ligands to GPCRs.

In 2011, 7α,25-diHC and 7α,26-diHC were discovered to be ligands to GPCR183 known EBI2 [7,8]. Both 7α,25-diHC and 7α,26-diHC act as chemo-attractants to cells expressing EBI2, directing cell migration. Mice deficient in EBI2 or in CH25H fail to position activated B cells within the spleen to the outer follicle and mount a reduced plasma cell response after an immune challenge, indicating a role for 7α,25-diHC in the immune response [7]. Numerous follow-up studies have implicated oxysterols and EBI2 in inflammatory disorders, including neuroinflammation [117,118]. However, 25−HC, one of the precursor oxysterol to 7α,25-diHC, appears to have pro- and anti-inflammatory properties in different situations. Cyster, Russell and co-workers have defined an anti-inflammatory activity of 25−HC through prevention of AIM2 inflammasome activation in macrophages [39,40]. They showed that this is through inhibition of the SREBP-2 pathway and reduced synthesis of cholesterol [40]. Their results are consistent with data from Crick et al who showed that levels of 25−HC are reduced in plasma from patients with the inflammatory disorder multiple sclerosis [119]. On-the-other hand Vigne et al showed 25−HC acting through LXR to dampen the response of regulatory T cells, resulting in a pro-inflammatory response [120]. Clearly more work is required to understand how 25−HC, 7α,25-diHC and their synthetic enzymes act under different conditions in different types of immune cell.

SMO is a Class F GPCR [121]. It is a key component of the Hh signalling pathway required for proper cell differentiation and malfunction of the pathway leads to basal cell carcinoma [122]. SLOS phenocopies a defective Hh signalling pathway presenting with dysmorphology [82]. Oxysterols have been known for many years to be modulators of the Hh pathway through binding to SMO [9,123]. As discussed above, these include a wide range of structures ranging from the side-chain oxysterols 24S,25-EC and 20S−HC to B-ring oxysterols 7β,26-diHC and 26H,7−OC through to 3β,5α-dihydroxycholest-7-en-6-one [[9], [10], [11], [12]]. Interestingly, 3β,5α-dihydroxycholest-7-en-6-one appears to act in a different way to the side-chain and other B-ring oxysterols in that it binds to a different site on SMO and inhibits rather than activates the Hh pathway [11]. 3β,5α-Dihydroxycholest-7-en-6-one and the other B-ring oxysterols have been identified in plasma from SLOS patients, further linking dysfunctional Hh signalling to this disorder [113].

In summary, oxysterols were once regarded as uninteresting intermediates in bile acid and steroid hormone biosynthesis. That view has changed with compelling evidence for their biological activities including acting as lipid mediators in physiologic and pathologic conditions. The observed modifications of oxysterol metabolite profiles in numerous diseases (age related diseases, neurological diseases, etc) could be of value in identifying new pharmacological targets and in development of efficient treatments.

## References

[1] Schroepfer G.J. (2000). Oxysterols: modulators of cholesterol metabolism and other processes. Physiol. Rev..

[2] Javitt N.B. (2004). Oxysteroids: a new class of steroids with autocrine and paracrine functions. Trends Endocrinol. Metab..

[3] Lehmann J.M., Kliewer S.A., Moore L.B., Smith-Oliver T.A., Oliver B.B., Su J.L., Sundseth S.S., Winegar D.A., Blanchard D.E., Spencer T.A., Willson T.M. (1997). Activation of the nuclear receptor LXR by oxysterols defines a new hormone response pathway. J. Biol. Chem..

[4] Umetani M., Domoto H., Gormley A.K., Yuhanna I.S., Cummins C.L., Javitt N.B., Korach K.S., Shaul P.W., Mangelsdorf D.J. (2007). 27-hydroxycholesterol is an endogenous SERM that inhibits the cardiovascular effects of estrogen. Nat. Med..

[5] Soroosh P., Wu J., Xue X., Song J., Sutton S.W., Sablad M., Yu J., Nelen M.I., Liu X., Castro G., Luna R., Crawford S., Banie H., Dandridge R.A., Deng X., Bittner A., Kuei C., Tootoonchi M., Rozenkrants N., Herman K., Gao J., Yang X.V., Sachen K., Ngo K., Fung-Leung W.P., Nguyen S., de Leon-Tabaldo A., Blevitt J., Zhang Y., Cummings M.D., Rao T., Mani N.S., Liu C., McKinnon M., Milla M.E., Fourie A.M., Sun S. (2014). Oxysterols are agonist ligands of RORgammat and drive Th17 cell differentiation. Proc. Natl. Acad. Sci. U. S. A..

[6] Voisin M., de Medina P., Mallinger A., Dalenc F., Huc-Claustre E., Leignadier J., Serhan N., Soules R., Segala G., Mougel A., Noguer E., Mhamdi L., Bacquie E., Iuliano L., Zerbinati C., Lacroix-Triki M., Chaltiel L., Filleron T., Cavailles V., Al Saati T., Rochaix P., Duprez-Paumier R., Franchet C., Ligat L., Lopez F., Record M., Poirot M., Silvente-Poirot S. (2017). Identification of a tumor-promoter cholesterol metabolite in human breast cancers acting through the glucocorticoid receptor. Proc. Natl. Acad. Sci. U. S. A..

[7] Hannedouche S., Zhang J., Yi T., Shen W., Nguyen D., Pereira J.P., Guerini D., Baumgarten B.U., Roggo S., Wen B., Knochenmuss R., Noel S., Gessier F., Kelly L.M., Vanek M., Laurent S., Preuss I., Miault C., Christen I., Karuna R., Li W., Koo D.I., Suply T., Schmedt C., Peters E.C., Falchetto R., Katopodis A., Spanka C., Roy M.O., Detheux M., Chen Y.A., Schultz P.G., Cho C.Y., Seuwen K., Cyster J.G., Sailer A.W. (2011). Oxysterols direct immune cell migration via EBI2. Nature.

[8] Liu C., Yang X.V., Wu J., Kuei C., Mani N.S., Zhang L., Yu J., Sutton S.W., Qin N., Banie H., Karlsson L., Sun S., Lovenberg T.W. (2011). Oxysterols direct B-cell migration through EBI2. Nature.

[9] Nachtergaele S., Mydock L.K., Krishnan K., Rammohan J., Schlesinger P.H., Covey D.F., Rohatgi R. (2012). Oxysterols are allosteric activators of the oncoprotein smoothened. Nat. Chem. Biol..

[10] Myers B.R., Sever N., Chong Y.C., Kim J., Belani J.D., Rychnovsky S., Bazan J.F., Beachy P.A. (2013). Hedgehog pathway modulation by multiple lipid binding sites on the smoothened effector of signal response. Dev. Cell.

[11] Sever N., Mann R.K., Xu L., Snell W.J., Hernandez-Lara C.I., Porter N.A., Beachy P.A. (2016). Endogenous B-ring oxysterols inhibit the hedgehog component smoothened in a manner distinct from cyclopamine or side-chain oxysterols. Proc. Natl. Acad. Sci. U. S. A..

[12] Raleigh D.R., Sever N., Choksi P.K., Sigg M.A., Hines K.M., Thompson B.M., Elnatan D., Jaishankar P., Bisignano P., Garcia-Gonzalo F.R., Krup A.L., Eberl M., Byrne E.F.X., Siebold C., Wong S.Y., Renslo A.R., Grabe M., McDonald J.G., Xu L., Beachy P.A., Reiter J.F. (2018). Cilia-associated oxysterols activate smoothened. Mol. Cell.

[13] Sun M.Y., Linsenbardt A.J., Emnett C.M., Eisenman L.N., Izumi Y., Zorumski C.F., Mennerick S. (2016). 24(S)-hydroxycholesterol as a modulator of neuronal signaling and survival. Neuroscientist.

[14] Radhakrishnan A., Ikeda Y., Kwon H.J., Brown M.S., Goldstein J.L. (2007). Sterol-regulated transport of SREBPs from endoplasmic reticulum to golgi: oxysterols block transport by binding to insig. Proc. Natl. Acad. Sci. U. S. A..

[15] Russell D.W. (2003). The enzymes, regulation, and genetics of bile acid synthesis. Annu. Rev. Biochem.

[16] Murphy R.C., Johnson K.M. (2008). Cholesterol, reactive oxygen species, and the formation of biologically active mediators. J. Biol. Chem..

[17] Xu L., Korade Z., Rosado D.A., Mirnics K., Porter N.A. (2013). Metabolism of oxysterols derived from nonenzymatic oxidation of 7-dehydrocholesterol in cells. J. Lipid Res..

[18] Zerbinati C., Iuliano L. (2017). Cholesterol and related sterols autoxidation. Free Radic. Biol. Med..

[19] Lund E.G., Kerr T.A., Sakai J., Li W.P., Russell D.W. (1998). cDNA cloning of mouse and human cholesterol 25-hydroxylases, polytopic membrane proteins that synthesize a potent oxysterol regulator of lipid metabolism. J. Biol. Chem..

[20] Ishibashi S., Schwarz M., Frykman P.K., Herz J., Russell D.W. (1996). Disruption of cholesterol 7alpha-hydroxylase gene in mice. I. Postnatal lethality reversed by bile acid and vitamin supplementation. J. Biol. Chem..

[21] Fagerberg L., Hallstrom B.M., Oksvold P., Kampf C., Djureinovic D., Odeberg J., Habuka M., Tahmasebpoor S., Danielsson A., Edlund K., Asplund A., Sjostedt E., Lundberg E., Szigyarto C.A., Skogs M., Takanen J.O., Berling H., Tegel H., Mulder J., Nilsson P., Schwenk J.M., Lindskog C., Danielsson F., Mardinoglu A., Sivertsson A., von Feilitzen K., Forsberg M., Zwahlen M., Olsson I., Navani S., Huss M., Nielsen J., Ponten F., Uhlen M. (2014). Analysis of the human tissue-specific expression by genome-wide integration of transcriptomics and antibody-based proteomics. Mol. Cell. Proteomics.

[22] Yue F., Cheng Y., Breschi A., Vierstra J., Wu W., Ryba T., Sandstrom R., Ma Z., Davis C., Pope B.D., Shen Y., Pervouchine D.D., Djebali S., Thurman R.E., Kaul R., Rynes E., Kirilusha A., Marinov G.K., Williams B.A., Trout D., Amrhein H., Fisher-Aylor K., Antoshechkin I., DeSalvo G., See L.H., Fastuca M., Drenkow J., Zaleski C., Dobin A., Prieto P., Lagarde J., Bussotti G., Tanzer A., Denas O., Li K., Bender M.A., Zhang M., Byron R., Groudine M.T., McCleary D., Pham L., Ye Z., Kuan S., Edsall L., Wu Y.C., Rasmussen M.D., Bansal M.S., Kellis M., Keller C.A., Morrissey C.S., Mishra T., Jain D., Dogan N., Harris R.S., Cayting P., Kawli T., Boyle A.P., Euskirchen G., Kundaje A., Lin S., Lin Y., Jansen C., Malladi V.S., Cline M.S., Erickson D.T., Kirkup V.M., Learned K., Sloan C.A., Rosenbloom K.R., Lacerda de Sousa B., Beal K., Pignatelli M., Flicek P., Lian J., Kahveci T., Lee D., Kent W.J., Ramalho Santos M., Herrero J., Notredame C., Johnson A., Vong S., Lee K., Bates D., Neri F., Diegel M., Canfield T., Sabo P.J., Wilken M.S., Reh T.A., Giste E., Shafer A., Kutyavin T., Haugen E., Dunn D., Reynolds A.P., Neph S., Humbert R., Hansen R.S., De Bruijn M., Selleri L., Rudensky A., Josefowicz S., Samstein R., Eichler E.E., Orkin S.H., Levasseur D., Papayannopoulou T., Chang K.H., Skoultchi A., Gosh S., Disteche C., Treuting P., Wang Y., Weiss M.J., Blobel G.A., Cao X., Zhong S., Wang T., Good P.J., Lowdon R.F., Adams L.B., Zhou X.Q., Pazin M.J., Feingold E.A., Wold B., Taylor J., Mortazavi A., Weissman S.M., Stamatoyannopoulos J.A., Snyder M.P., Guigo R., Gingeras T.R., Gilbert D.M., Hardison R.C., Beer M.A., Ren B., Mouse E.C. (2014). A comparative encyclopedia of DNA elements in the mouse genome. Nature.

[23] Cali J.J., Russell D.W. (1991). Characterization of human sterol 27-hydroxylase. A mitochondrial cytochrome P-450 that catalyzes multiple oxidation reaction in bile acid biosynthesis. J. Biol. Chem..

[24] Shea H.C., Head D.D., Setchell K.D., Russell D.W. (2007). Analysis of HSD3B7 knockout mice reveals that a 3alpha-hydroxyl stereochemistry is required for bile acid function. Proc. Natl. Acad. Sci. U. S. A..

[25] Dussault I., Yoo H.D., Lin M., Wang E., Fan M., Batta A.K., Salen G., Erickson S.K., Forman B.M. (2003). Identification of an endogenous ligand that activates pregnane X receptor-mediated sterol clearance. Proc. Natl. Acad. Sci. U. S. A..

[26] Griffiths W.J., Crick P.J., Meljon A., Theofilopoulos S., Abdel-Khalik J., Yutuc E., Parker J.E., Kelly D.E., Kelly S.L., Arenas E., Wang Y. (2019). Additional pathways of sterol metabolism: evidence from analysis of Cyp27a1-/- mouse brain and plasma. Biochim. Biophys. Acta. Mol. Cell Biol. Lipids.

[27] Honda A., Salen G., Matsuzaki Y., Batta A.K., Xu G., Leitersdorf E., Tint G.S., Erickson S.K., Tanaka N., Shefer S. (2001). Side chain hydroxylations in bile acid biosynthesis catalyzed by CYP3A are markedly up-regulated in Cyp27-/- mice but not in cerebrotendinous xanthomatosis. J. Biol. Chem..

[28] Wang J., Greene S., Eriksson L.C., Rozell B., Reihner E., Einarsson C., Eggertsen G., Gafvels M. (2005). Human sterol 12a-hydroxylase (CYP8B1) is mainly expressed in hepatocytes in a homogenous pattern. Histochem. Cell Biol..

[29] Wang J., Olin M., Rozell B., Bjorkhem I., Einarsson C., Eggertsen G., Gafvels M. (2007). Differential hepatocellular zonation pattern of cholesterol 7alpha-hydroxylase (Cyp7a1) and sterol 12alpha-hydroxylase (Cyp8b1) in the mouse. Histochem. Cell Biol..

[30] Ferdinandusse S., Denis S., Overmars H., Van Eeckhoudt L., Van Veldhoven P.P., Duran M., Wanders R.J., Baes M. (2005). Developmental changes of bile acid composition and conjugation in L- and D-bifunctional protein single and double knockout mice. J. Biol. Chem..

[31] Ferdinandusse S., Denis S., Faust P.L., Wanders R.J. (2009). Bile acids: the role of peroxisomes. J. Lipid Res..

[32] Vaz F.M., Ferdinandusse S. (2017). Bile acid analysis in human disorders of bile acid biosynthesis. Mol. Aspects Med..

[33] Makishima M., Okamoto A.Y., Repa J.J., Tu H., Learned R.M., Luk A., Hull M.V., Lustig K.D., Mangelsdorf D.J., Shan B. (1999). Identification of a nuclear receptor for bile acids. Science.

[34] Parks D.J., Blanchard S.G., Bledsoe R.K., Chandra G., Consler T.G., Kliewer S.A., Stimmel J.B., Willson T.M., Zavacki A.M., Moore D.D., Lehmann J.M. (1999). Bile acids: natural ligands for an orphan nuclear receptor. Science.

[35] Lu T.T., Makishima M., Repa J.J., Schoonjans K., Kerr T.A., Auwerx J., Mangelsdorf D.J. (2000). Molecular basis for feedback regulation of bile acid synthesis by nuclear receptors. Mol. Cell.

[36] Bauman D.R., Bitmansour A.D., McDonald J.G., Thompson B.M., Liang G., Russell D.W. (2009). 25-hydroxycholesterol secreted by macrophages in response to Toll-like receptor activation suppresses immunoglobulin a production. Proc. Natl. Acad. Sci. U. S. A..

[37] Park K., Scott A.L. (2010). Cholesterol 25-hydroxylase production by dendritic cells and macrophages is regulated by type I interferons. J. Leukoc. Biol..

[38] Blanc M., Hsieh W.Y., Robertson K.A., Kropp K.A., Forster T., Shui G., Lacaze P., Watterson S., Griffiths S.J., Spann N.J., Meljon A., Talbot S., Krishnan K., Covey D.F., Wenk M.R., Craigon M., Ruzsics Z., Haas J., Angulo A., Griffiths W.J., Glass C.K., Wang Y., Ghazal P. (2013). The transcription factor STAT-1 couples macrophage synthesis of 25-hydroxycholesterol to the interferon antiviral response. Immunity.

[39] Reboldi A., Dang E.V., McDonald J.G., Liang G., Russell D.W., Cyster J.G. (2014). Inflammation. 25-hydroxycholesterol suppresses interleukin-1-driven inflammation downstream of type I interferon. Science.

[40] Dang E.V., McDonald J.G., Russell D.W., Cyster J.G. (2017). Oxysterol restraint of cholesterol synthesis prevents AIM2 inflammasome activation. Cell.

[41] Liu S.Y., Aliyari R., Chikere K., Li G., Marsden M.D., Smith J.K., Pernet O., Guo H., Nusbaum R., Zack J.A., Freiberg A.N., Su L., Lee B., Cheng G. (2013). Interferon-inducible cholesterol-25-hydroxylase broadly inhibits viral entry by production of 25-hydroxycholesterol. Immunity.

[42] Goenka A., Ghosh A., Dixon S., Griffiths W.J., Hughes S.M., Newman W.G., Urquhart J., Wang Y., Wynn R.F., Hussell T., Jones S.A., Arkwright P.D. (2017). Susceptibility to BCG abscess associated with deletion of two cholesterol metabolism genes: lysosomal acid lipase and cholesterol 25-hydroxylase. UKPIN Conference.

[43] Honda A., Miyazaki T., Ikegami T., Iwamoto J., Maeda T., Hirayama T., Saito Y., Teramoto T., Matsuzaki Y. (2011). Cholesterol 25-hydroxylation activity of CYP3A. J. Lipid Res..

[44] Li-Hawkins J., Lund E.G., Turley S.D., Russell D.W. (2000). Disruption of the oxysterol 7alpha-hydroxylase gene in mice. J. Biol. Chem..

[45] Setchell K.D., Schwarz M., O’Connell N.C., Lund E.G., Davis D.L., Lathe R., Thompson H.R., Weslie Tyson R., Sokol R.J., Russell D.W. (1998). Identification of a new inborn error in bile acid synthesis: mutation of the oxysterol 7alpha-hydroxylase gene causes severe neonatal liver disease. J. Clin. Invest..

[46] Schols L., Rattay T.W., Martus P., Meisner C., Baets J., Fischer I., Jagle C., Fraidakis M.J., Martinuzzi A., Saute J.A., Scarlato M., Antenora A., Stendel C., Hoflinger P., Lourenco C.M., Abreu L., Smets K., Paucar M., Deconinck T., Bis D.M., Wiethoff S., Bauer P., Arnoldi A., Marques W., Jardim L.B., Hauser S., Criscuolo C., Filla A., Zuchner S., Bassi M.T., Klopstock T., De Jonghe P., Bjorkhem I., Schule R. (2017). Hereditary spastic paraplegia type 5: natural history, biomarkers and a randomized controlled trial. Brain.

[47] Duane W.C., Pooler P.A., Hamilton J.N. (1988). Bile acid synthesis in man. In vivo activity of the 25-hydroxylation pathway. J. Clin. Invest..

[48] Griffiths W.J., Abdel-Khalik J., Yutuc E., Roman G., Warner M., Gustafsson J.A., Wang Y. (2018). Concentrations of bile acid precursors in cerebrospinal fluid of Alzheimer’s disease patients. Free Radic. Biol. Med..

[49] Abdel-Khalik J., Crick P.J., Yutuc E., DeBarber A.E., Duell P.B., Steiner R.D., Laina I., Wang Y., Griffiths W.J. (2018). Identification of 7alpha,24-dihydroxy-3-oxocholest-4-en-26-oic and 7alpha,25-dihydroxy-3-oxocholest-4-en-26-oic acids in human cerebrospinal fluid and plasma. Biochimie.

[50] Lutjohann D., Breuer O., Ahlborg G., Nennesmo I., Siden A., Diczfalusy U., Bjorkhem I. (1996). Cholesterol homeostasis in human brain: evidence for an age-dependent flux of 24S-hydroxycholesterol from the brain into the circulation. Proc. Natl. Acad. Sci. U. S. A..

[51] Lund E.G., Guileyardo J.M., Russell D.W. (1999). cDNA cloning of cholesterol 24-hydroxylase, a mediator of cholesterol homeostasis in the brain. Proc. Natl. Acad. Sci. U. S. A..

[52] Saeed A.A., Genove G., Li T., Lutjohann D., Olin M., Mast N., Pikuleva I.A., Crick P., Wang Y., Griffiths W., Betsholtz C., Bjorkhem I. (2014). Effects of a disrupted blood-brain barrier on cholesterol homeostasis in the brain. J. Biol. Chem..

[53] Li-Hawkins J., Lund E.G., Bronson A.D., Russell D.W. (2000). Expression cloning of an oxysterol 7alpha-hydroxylase selective for 24-hydroxycholesterol. J. Biol. Chem..

[54] Meljon A., Crick P.J., Yutuc E., Yau J.L., Seckl J.R., Theofilopoulos S., Arenas E., Wang Y., Griffiths W.J. (2019). Mining for oxysterols in Cyp7b1(-/-) mouse brain and plasma: relevance to spastic paraplegia type 5. Biomolecules.

[55] Griffiths W.J., Yutuc E., Abdel-Khalik J., Crick P.J., Hearn T., Dickson A., Bigger B.W., Hoi-Yee Wu T., Goenka A., Ghosh A., Jones S.A., Covey D.F., Ory D.S., Wang Y. (2019). Metabolism of Non-enzymatically derived oxysterols: clues from sterol metabolic disorders. Free Radic. Biol. Med..

[56] Bjorkhem I. (2013). Cerebrotendinous xanthomatosis. Curr. Opin. Lipidol..

[57] Rosen H., Reshef A., Maeda N., Lippoldt A., Shpizen S., Triger L., Eggertsen G., Bjorkhem I., Leitersdorf E. (1998). Markedly reduced bile acid synthesis but maintained levels of cholesterol and vitamin D metabolites in mice with disrupted sterol 27-hydroxylase gene. J. Biol. Chem..

[58] Song C., Liao S. (2000). Cholestenoic acid is a naturally occurring ligand for liver X receptor alpha. Endocrinology.

[59] Theofilopoulos S., Griffiths W.J., Crick P.J., Yang S., Meljon A., Ogundare M., Kitambi S.S., Lockhart A., Tuschl K., Clayton P.T., Morris A.A., Martinez A., Reddy M.A., Martinuzzi A., Bassi M.T., Honda A., Mizuochi T., Kimura A., Nittono H., De Michele G., Carbone R., Criscuolo C., Yau J.L., Seckl J.R., Schule R., Schols L., Sailer A.W., Kuhle J., Fraidakis M.J., Gustafsson J.A., Steffensen K.R., Bjorkhem I., Ernfors P., Sjovall J., Arenas E., Wang Y. (2014). Cholestenoic acids regulate motor neuron survival via liver X receptors. J. Clin. Invest..

[60] Ogundare M., Theofilopoulos S., Lockhart A., Hall L.J., Arenas E., Sjovall J., Brenton A.G., Wang Y., Griffiths W.J. (2010). Cerebrospinal fluid steroidomics: are bioactive bile acids present in brain?. J. Biol. Chem..

[61] Fu X., Menke J.G., Chen Y., Zhou G., MacNaul K.L., Wright S.D., Sparrow C.P., Lund E.G. (2001). 27-hydroxycholesterol is an endogenous ligand for liver X receptor in cholesterol-loaded cells. J. Biol. Chem..

[62] DuSell C.D., Umetani M., Shaul P.W., Mangelsdorf D.J., McDonnell D.P. (2008). 27-hydroxycholesterol is an endogenous selective estrogen receptor modulator. Mol. Endocrinol..

[63] He S., Nelson E.R. (2017). 27-hydroxycholesterol, an endogenous selective estrogen receptor modulator. Maturitas.

[64] Ferdinandusse S., Houten S.M. (2006). Peroxisomes and bile acid biosynthesis. Biochim. Biophys. Acta.

[65] Steinberg S.J., Dodt G., Raymond G.V., Braverman N.E., Moser A.B., Moser H.W. (2006). Peroxisome biogenesis disorders. Biochim. Biophys. Acta.

[66] Ferdinandusse S., Denis S., Clayton P.T., Graham A., Rees J.E., Allen J.T., McLean B.N., Brown A.Y., Vreken P., Waterham H.R., Wanders R.J. (2000). Mutations in the gene encoding peroxisomal alpha-methylacyl-CoA racemase cause adult-onset sensory motor neuropathy. Nat. Genet..

[67] Autio K.J., Schmitz W., Nair R.R., Selkala E.M., Sormunen R.T., Miinalainen I.J., Crick P.J., Wang Y., Griffiths W.J., Reddy J.K., Baes M., Hiltunen J.K. (2014). Role of AMACR (alpha-methylacyl-CoA racemase) and MFE-1 (peroxisomal multifunctional enzyme-1) in bile acid synthesis in mice. Biochem. J.

[68] Vilarinho S., Sari S., Mazzacuva F., Bilguvar K., Esendagli-Yilmaz G., Jain D., Akyol G., Dalgic B., Gunel M., Clayton P.T., Lifton R.P. (2016). ACOX2 deficiency: a disorder of bile acid synthesis with transaminase elevation, liver fibrosis, ataxia, and cognitive impairment. Proc. Natl. Acad. Sci. U. S. A..

[69] Hunt M.C., Solaas K., Kase B.F., Alexson S.E. (2002). Characterization of an acyl-coA thioesterase that functions as a major regulator of peroxisomal lipid metabolism. J. Biol. Chem..

[70] Hunt M.C., Siponen M.I., Alexson S.E. (2012). The emerging role of acyl-CoA thioesterases and acyltransferases in regulating peroxisomal lipid metabolism. Biochim. Biophys. Acta.

[71] Ferdinandusse S., Ylianttila M.S., Gloerich J., Koski M.K., Oostheim W., Waterham H.R., Hiltunen J.K., Wanders R.J., Glumoff T. (2006). Mutational spectrum of D-bifunctional protein deficiency and structure-based genotype-phenotype analysis. Am. J. Hum. Genet..

[72] Ferdinandusse S., Kostopoulos P., Denis S., Rusch H., Overmars H., Dillmann U., Reith W., Haas D., Wanders R.J., Duran M., Marziniak M. (2006). Mutations in the gene encoding peroxisomal sterol carrier protein X (SCPx) cause leukencephalopathy with dystonia and motor neuropathy. Am. J. Hum. Genet..

[73] Setchell K.D., Heubi J.E., Shah S., Lavine J.E., Suskind D., Al-Edreesi M., Potter C., Russell D.W., O’Connell N.C., Wolfe B., Jha P., Zhang W., Bove K.E., Knisely A.S., Hofmann A.F., Rosenthal P., Bull L.N. (2013). Genetic defects in bile acid conjugation cause fat-soluble vitamin deficiency. Gastroenterology.

[74] Shinkyo R., Xu L., Tallman K.A., Cheng Q., Porter N.A., Guengerich F.P. (2011). Conversion of 7-dehydrocholesterol to 7-ketocholesterol is catalyzed by human cytochrome P450 7A1 and occurs by direct oxidation without an epoxide intermediate. J. Biol. Chem..

[75] Schweizer R.A., Zurcher M., Balazs Z., Dick B., Odermatt A. (2004). Rapid hepatic metabolism of 7-ketocholesterol by 11beta-hydroxysteroid dehydrogenase type 1: species-specific differences between the rat, human, and hamster enzyme. J. Biol. Chem..

[76] Hult M., Elleby B., Shafqat N., Svensson S., Rane A., Jornvall H., Abrahmsen L., Oppermann U. (2004). Human and rodent type 1 11beta-hydroxysteroid dehydrogenases are 7beta-hydroxycholesterol dehydrogenases involved in oxysterol metabolism. Cell. Mol. Life Sci..

[77] Mitic T., Shave S., Semjonous N., McNae I., Cobice D.F., Lavery G.G., Webster S.P., Hadoke P.W., Walker B.R., Andrew R. (2013). 11beta-hydroxysteroid dehydrogenase type 1 contributes to the balance between 7-keto- and 7-hydroxy-oxysterols in vivo. Biochem. Pharmacol..

[78] Griffiths W.J., Yutuc E., Abdel-Khalik J., Wang Y. (2017). Metabolism of Non-Enzymatically Derived Oxysterols, 7th ENOR SYMPOSIUM: Oxysterols and Sterol Derivatives in Health and Disease.

[79] Wang Y., Griffiths W.J. (2018). Unravelling new pathways of sterol metabolism: lessons learned from in-born errors and cancer. Curr. Opin. Clin. Nutr. Metab. Care.

[80] Beck K.R., Kanagaratnam S., Kratschmar D.V., Birk J., Yamaguchi H., Sailer A.W., Seuwen K., Odermatt A. (2019). Enzymatic interconversion of the oxysterols 7beta,25-dihydroxycholesterol and 7-keto,25-hydroxycholesterol by 11beta-hydroxysteroid dehydrogenase type 1 and 2. J. Steroid Biochem. Mol. Biol..

[81] Beck K.R., Inderbinen S.G., Kanagaratnam S., Kratschmar D.V., Jetten A.M., Yamaguchi H., Odermatt A. (2019). 11beta-hydroxysteroid dehydrogenases control access of 7beta,27-dihydroxycholesterol to retinoid-related orphan receptor gamma. J. Lipid Res..

[82] Kelley R.L., Roessler E., Hennekam R.C., Feldman G.L., Kosaki K., Jones M.C., Palumbos J.C., Muenke M. (1996). Holoprosencephaly in RSH/Smith-lemli-opitz syndrome: does abnormal cholesterol metabolism affect the function of sonic hedgehog?. Am. J. Med. Genet..

[83] Mazzacuva F., Mills P., Mills K., Camuzeaux S., Gissen P., Nicoli E.R., Wassif C., Te Vruchte D., Porter F.D., Maekawa M., Mano N., Iida T., Platt F., Clayton P.T. (2016). Identification of novel bile acids as biomarkers for the early diagnosis of niemann-pick C disease. FEBS Lett..

[84] Vance J.E., Karten B. (2014). Niemann-pick C disease and mobilization of lysosomal cholesterol by cyclodextrin. J. Lipid Res..

[85] Miller W.L., Bose H.S. (2011). Early steps in steroidogenesis: intracellular cholesterol trafficking. J. Lipid Res..

[86] Vanier M.T., Gissen P., Bauer P., Coll M.J., Burlina A., Hendriksz C.J., Latour P., Goizet C., Welford R.W., Marquardt T., Kolb S.A. (2016). Diagnostic tests for niemann-pick disease type C (NP-C): a critical review. Mol. Genet. Metab..

[87] Silvente-Poirot S., Poirot M. (2012). Cholesterol epoxide hydrolase and cancer. Curr. Opin. Pharmacol..

[88] Segala G., David M., de Medina P., Poirot M.C., Serhan N., Vergez F., Mougel A., Saland E., Carayon K., Leignadier J., Caron N., Voisin M., Cherier J., Ligat L., Lopez F., Noguer E., Rives A., Payre B., Saati T.A., Lamaziere A., Despres G., Lobaccaro J.M., Baron S., Demur C., de Toni F., Larrue C., Boutzen H., Thomas F., Sarry J.E., Tosolini M., Picard D., Record M., Recher C., Poirot M., Silvente-Poirot S. (2017). Dendrogenin a drives LXR to trigger lethal autophagy in cancers. Nat. Commun..

[89] Poirot M., Silvente-Poirot S. (2018). The tumor-suppressor cholesterol metabolite, dendrogenin a, is a new class of LXR modulator activating lethal autophagy in cancers. Biochem. Pharmacol..

[90] Jiang X., Sidhu R., Mydock-McGrane L., Hsu F.F., Covey D.F., Scherrer D.E., Earley B., Gale S.E., Farhat N.Y., Porter F.D., Dietzen D.J., Orsini J.J., Berry-Kravis E., Zhang X., Reunert J., Marquardt T., Runz H., Giugliani R., Schaffer J.E., Ory D.S. (2016). Development of a bile acid-based newborn screen for niemann-pick disease type C. Sci. Transl. Med..

[91] Griffiths W.J., Gilmore I., Yutuc E., Abdel-Khalik J., Crick P.J., Hearn T., Dickson A., Bigger B.W., Wu T.H., Goenka A., Ghosh A., Jones S.A., Wang Y. (2018). Identification of unusual oxysterols and bile acids with 7-oxo or 3beta,5alpha,6beta-trihydroxy functions in human plasma by charge-tagging mass spectrometry with multistage fragmentation. J. Lipid Res..

[92] Alvelius G., Hjalmarson O., Griffiths W.J., Bjorkhem I., Sjovall J. (2001). Identification of unusual 7-oxygenated bile acid sulfates in a patient with niemann-pick disease, type C. J. Lipid Res..

[93] Natowicz M.R., Evans J.E. (1994). Abnormal bile acids in the Smith-lemli-opitz syndrome. Am. J. Med. Genet..

[94] Lyons M.A., Samman S., Gatto L., Brown A.J. (1999). Rapid hepatic metabolism of 7-ketocholesterol in vivo: implications for dietary oxysterols. J. Lipid Res..

[95] Lyons M.A., Brown A.J. (2001). Metabolism of an oxysterol, 7-ketocholesterol, by sterol 27-hydroxylase in HepG2 cells. Lipids.

[96] Nelson J.A., Steckbeck S.R., Spencer T.A. (1981). Biosynthesis of 24,25-epoxycholesterol from squalene 2,3;22,23-dioxide. J. Biol. Chem..

[97] Gill S., Chow R., Brown A.J. (2008). Sterol regulators of cholesterol homeostasis and beyond: the oxysterol hypothesis revisited and revised. Prog. Lipid Res..

[98] Goyal S., Xiao Y., Porter N.A., Xu L., Guengerich F.P. (2014). Oxidation of 7-dehydrocholesterol and desmosterol by human cytochrome P450 46A1. J. Lipid Res..

[99] Theofilopoulos S., Abreu de Oliveira W.A., Yang S., Yutuc E., Saeed A., Abdel-Khalik J., Ullgren A., Cedazo-Minguez A., Bjorkhem I., Wang Y., Griffiths W.J., Arenas E. (2019). 24(S),25-epoxycholesterol and cholesterol 24S-hydroxylase (CYP46A1) overexpression promote midbrain dopaminergic neurogenesis in vivo. J. Biol. Chem..

[100] Adams C.M., Reitz J., De Brabander J.K., Feramisco J.D., Li L., Brown M.S., Goldstein J.L. (2004). Cholesterol and 25-hydroxycholesterol inhibit activation of SREBPs by different mechanisms, both involving SCAP and insigs. J. Biol. Chem..

[101] Qi X., Liu H., Thompson B., McDonald J., Zhang C., Li X. (2019). Cryo-EM structure of oxysterol-bound human smoothened coupled to a heterotrimeric Gi. Nature.

[102] Theofilopoulos S., Wang Y., Kitambi S.S., Sacchetti P., Sousa K.M., Bodin K., Kirk J., Salto C., Gustafsson M., Toledo E.M., Karu K., Gustafsson J.A., Steffensen K.R., Ernfors P., Sjovall J., Griffiths W.J., Arenas E. (2013). Brain endogenous liver X receptor ligands selectively promote midbrain neurogenesis. Nat. Chem. Biol..

[103] Wang Y., Karu K., Meljon A., Turton J., Yau J.L., Seckl J.R., Wang Y., Griffiths W.J. (2014). 24S,25-epoxycholesterol in mouse and rat brain. Biochem. Biophys. Res. Commun..

[104] Roberg-Larsen H., Strand M.F., Krauss S., Wilson S.R. (2014). Metabolites in vertebrate hedgehog signaling. Biochem. Biophys. Res. Commun..

[105] Lin Y.Y., Welch M., Lieberman S. (2003). The detection of 20S-hydroxycholesterol in extracts of rat brains and human placenta by a gas chromatograph/mass spectrometry technique. J. Steroid Biochem. Mol. Biol..

[106] Griffiths W.J., Yutuc E., Angelini R., Baumert M., Mast N., Pikuleva I., Newton J., Clench M.R., Howell O., Wang Y. (2018). Imaging Oxysterols in Mouse Brain by On-Tissue Derivatisation-Robotic Liquid Micro-Extraction Surface Analysis-Liquid Chromatography Mass Spectrometry.

[107] Wechsler A., Brafman A., Shafir M., Heverin M., Gottlieb H., Damari G., Gozlan-Kelner S., Spivak I., Moshkin O., Fridman E., Becker Y., Skaliter R., Einat P., Faerman A., Bjorkhem I., Feinstein E. (2003). Generation of viable cholesterol-free mice. Science.

[108] Heverin M., Meaney S., Brafman A., Shafir M., Olin M., Shafaati M., von Bahr S., Larsson L., Lovgren-Sandblom A., Diczfalusy U., Parini P., Feinstein E., Bjorkhem I. (2007). Studies on the cholesterol-free mouse: strong activation of LXR-regulated hepatic genes when replacing cholesterol with desmosterol. Arterioscler. Thromb. Vasc. Biol..

[109] Yang C., McDonald J.G., Patel A., Zhang Y., Umetani M., Xu F., Westover E.J., Covey D.F., Mangelsdorf D.J., Cohen J.C., Hobbs H.H. (2006). Sterol intermediates from cholesterol biosynthetic pathway as liver X receptor ligands. J. Biol. Chem..

[110] Saini R., Kataeva O., Schmidt A.W., Wang Y., Meljon A., Griffiths W.J., Knolker H.J. (2013). Synthesis and biological activity of (24E)- and (24Z)-26-hydroxydesmosterol. Bioorg. Med. Chem..

[111] Xu L., Porter N.A. (2015). Free radical oxidation of cholesterol and its precursors: implications in cholesterol biosynthesis disorders. Free Radic. Res..

[112] Bjorkhem I., Diczfalusy U., Lovgren-Sandblom A., Starck L., Jonsson M., Tallman K., Schirmer H., Ousager L.B., Crick P.J., Wang Y., Griffiths W.J., Guengerich F.P. (2014). On the formation of 7-ketocholesterol from 7-dehydrocholesterol in patients with CTX and SLO. J. Lipid Res..

[113] Griffiths W.J., Abdel-Khalik J., Crick P.J., Ogundare M., Shackleton C.H., Tuschl K., Kwok M.K., Bigger B.W., Morris A.A., Honda A., Xu L., Porter N.A., Bjorkhem I., Clayton P.T., Wang Y. (2017). Sterols and oxysterols in plasma from Smith-lemli-opitz syndrome patients. J. Steroid Biochem. Mol. Biol..

[114] Wassif C.A., Yu J., Cui J., Porter F.D., Javitt N.B. (2003). 27-hydroxylation of 7- and 8-dehydrocholesterol in Smith-lemli-opitz syndrome: a novel metabolic pathway. Steroids.

[115] Endo-Umeda K., Yasuda K., Sugita K., Honda A., Ohta M., Ishikawa M., Hashimoto Y., Sakaki T., Makishima M. (2014). 7-dehydrocholesterol metabolites produced by sterol 27-hydroxylase (CYP27A1) modulate liver X receptor activity. J. Steroid Biochem. Mol. Biol..

[116] Lamberson C.R., Muchalski H., McDuffee K.B., Tallman K.A., Xu L., Porter N.A. (2017). Propagation rate constants for the peroxidation of sterols on the biosynthetic pathway to cholesterol. Chem. Phys. Lipids.

[117] Clottu A.S., Mathias A., Sailer A.W., Schluep M., Seebach J.D., Du Pasquier R., Pot C. (2017). EBI2 expression and function: robust in memory lymphocytes and increased by natalizumab in multiple sclerosis. Cell Rep.

[118] Mutemberezi V., Buisseret B., Masquelier J., Guillemot-Legris O., Alhouayek M., Muccioli G.G. (2018). Oxysterol levels and metabolism in the course of neuroinflammation: insights from in vitro and in vivo models. J Neuroinflammation.

[119] Crick P.J., Griffiths W.J., Zhang J., Beibel M., Abdel-Khalik J., Kuhle J., Sailer A.W., Wang Y. (2017). Reduced plasma levels of 25-hydroxycholesterol and increased cerebrospinal fluid levels of bile acid precursors in multiple sclerosis patients. Mol. Neurobiol..

[120] Vigne S., Chalmin F., Duc D., Clottu A.S., Apetoh L., Lobaccaro J.A., Christen I., Zhang J., Pot C. (2017). IL-27-induced type 1 regulatory T-cells produce oxysterols that constrain IL-10 production. Front. Immunol..

[121] Ruiz-Gomez A., Molnar C., Holguin H., Mayor F., de Celis J.F. (2007). The cell biology of smo signalling and its relationships with GPCRs. Biochim. Biophys. Acta.

[122] Xie J., Murone M., Luoh S.M., Ryan A., Gu Q., Zhang C., Bonifas J.M., Lam C.W., Hynes M., Goddard A., Rosenthal A., Epstein E.H., de Sauvage F.J. (1998). Activating smoothened mutations in sporadic basal-cell carcinoma. Nature.

[123] Corcoran R.B., Scott M.P. (2006). Oxysterols stimulate sonic hedgehog signal transduction and proliferation of medulloblastoma cells. Proc. Natl. Acad. Sci. U. S. A..

